# Role of bile salt in regulating Mcl-1 phosphorylation and chemoresistance in hepatocellular carcinoma cells

**DOI:** 10.1186/1476-4598-10-44

**Published:** 2011-04-20

**Authors:** Mingmei Liao, Jinfeng Zhao, Ton Wang, Jinghua Duan, Yangde Zhang, Xingming Deng

**Affiliations:** 1 National Hepatobiliary & Enteric Surgery Research Center, Department of Surgery, Xiangya Hospital, Central South University, Changsha, Hunan 410008, P.R. China; 2 Department of Radiation Oncology, Emory University School of Medicine and Winship Cancer Institute of Emory University, Atlanta, GA 30322, USA

## Abstract

**Background:**

Glycochenodeoxycholate (GCDA) is one of the major human bile salts. Bile salts stimulate cell survival and proliferation through the mitogen-activated protein kinase, but the downstream signaling mechanism(s) remains enigmatic. Mcl-1 is an antiapoptotic molecule of the Bcl2 family that is extensively overexpressed in tumor tissues of patients with hepatocellular carcinoma (HCC).

**Results:**

Here we found that exposure of HepG2 cells to GCDA results in activation of ERK1 and ERK2 and phosphorylation of Mcl-1 in a PD98059 (MEK inhibitor)-sensitive manner. GCDA stimulates Mcl-1 phosphorylation in cells expressing WT but not T163A Mcl-1 mutant, indicating that GCDA-induced Mcl-1 phosphorylation occurs exclusively at the T163 site in its PEST region. GCDA-induced Mcl-1 phosphorylation at T163 enhances the half-life of Mcl-1. Treatment of HepG2 cells with GCDA facilitates Mcl-1 dissociation from Mule (a physiological Mcl-1 ubiquitin E3 ligase). Specific depletion of Mcl-1 from HepG2 cells by RNA interference increases sensitivity of HepG2 cells to chemotherapeutic drugs (*i.e*. cisplatin and irinotecan). In addition to activation of the ERK/Mcl-1 survival pathway, GCDA can also induce dose-dependent apurinic/apyrimidinic (AP) sites of DNA lesions, which may partially neutralize its survival activity.

**Conclusion:**

Our findings suggest that bile salt may function as a survival agonist and/or potential carcinogen in the development of HCC. Molecular approaches that inactivate Mcl-1 by blocking its T163 phosphorylation may represent new strategies for treatment of HCC.

## Background

Hepatocellular carcinoma (HCC) is the 5^th ^most common cancer worldwide with approximately 564,000 new cases diagnosed every year [1]. Patients with HCC have poor prognosis, with few treatment options available [2]. Therefore, the development of novel strategies by identifying key targets at a molecular level is critical to cure HCC. Recent studies suggest that the aggressiveness, responsiveness to therapy and prognosis of HCC are closely linked to the Bcl-2 family members [1,3].

The Bcl2 family members have homology clustered within four conserved Bcl2 homology (BH) domains: BH1, BH2, BH3 and BH4, with only the antiapoptotic proteins, Bcl2, Bcl-XL, Bcl-w and A1, bearing the NH_2_-terminal BH4 domain [4]. In contrast, Mcl-1 has a helical BH4-like domain which is located between the PEST region and the BH3 domain [5]. The proapoptotic family members can be divided into two subgroups based on the presence of BH domains: the BH123 multidomain proteins *(i.e*. Bax and Bak) and the BH3-only molecules [6-8]. Recent studies suggest that there are two different subgroups in the BH3-only members. One group, including Bid and Bim, can function both directly to bind and activate Bax as well as indirectly to counteract the inhibition of Bax or Bak by antiapoptotic members including Bcl2 and Bcl-XL. Other BH3-only proteins (*i.e*. Bad, Bik, Noxa and PUMA) lack the ability to directly activate Bax but can oppose the action of antiapoptotic family members. Thus, both direct and indirect functions of BH3-only proteins may initiate apoptosis via selective interaction of its BH3 domain with an extended hydrophobic groove on the antiapoptotic Bcl2-like proteins and/or facilitate a conformational change in the multidomain proapoptotic proteins (*i.e*. Bax and Bak), which induce a death effect by promoting their insertion into mitochondrial membranes and oligomerization [6-8]. Bcl2 and related antiapoptotic proteins block the progression of a death signal by preventing Bax/Bak oligomerization [9].

Mcl-1 is a major antiapoptotic member of the Bcl2 family, which is crucial for liver development and hepatocellular homeostasis [10,11]. Mcl-1 is also an oncoprotein that promotes the development of cancer [12,13]. Importantly, Mcl-1 is overexpressed in about 50% of HCC patients [1], suggesting that Mcl-1 is a potential therapeutic target for some patients with HCC. In contrast to Bcl2 and Bcl-XL, Mcl-1 is rapidly inducible with a shorter half-life and seems to be more widely expressed in HCC [1,9,14]. Mcl-1 is mainly localized to the outer mitochondrial membrane via its C-terminal TM domain [15,16]. Several residues, including serine (S) 64, threonine (T) 92, S155, S159 and T163, have been identified as the potential phosphorylation sites following various stimuli [15,17-19]. However, phosphorylation of Mcl-1 at different site(s) distinctly regulates Mcl-1 protein turnover and its anti-apoptotic function [17-19]. For example, ERK1/2-mediated T163 site phosphorylation of Mcl-1 prolongs the half-life of Mcl-1, which leads to its increased antiapoptotic function [12,18]. We have recently discovered that nicotine induces Mcl-1 phosphorylation at T163 in association with increased chemoresistance of human lung cancer cells [20]. In contrast, GSK-3β-mediated Mcl-1 phosphorylation at the S159 site facilitates Mcl-1 ubiquitination and degradation leading to decreased survival activity [19]. It has been suggested that the BH3-only protein Bim can directly activate Bak, leading to mitochondrial dysfunction and apoptosis [6]. Thus, Mcl-1 may exert its antiapoptotic function via direct interaction with Bim leading to suppression of Bim-triggered Bak activation.

Bile is produced by the liver and consists of phospholipids and bile salts forming mixed micelles. Bile salts constitute a major portion of bile and are secreted by hepatocytes into the canaliculi. Hepatocellular bile salt secretion is mediated by an ATP-binding cassette (ABC) transporter named bile salt export pump (BSEP), which drives and maintains enterohepatic circulation of bile salts [21]. Bile salts have carcinogenic roles because bile salts not only induce DNA damage but also stimulate cell survival and proliferation [22-25]. Thus, accumulation of bile salts or bile acids in hepatocytes by some mechanisms may lead to chronic liver damage, proliferation or development of HCC [24-26]. Bile acid (*i.e*. deoxycholate) has been reported to enhance Mcl-1 expression through activation of the EGFR/Raf-1 signaling pathway in KMBC cells (a human cholangiocarcinoma cell line), which may participate in cholangiocyte carcinogenesis by inhibiting apoptosis [26]. It is currently unclear whether bile salts also regulate Mcl-1 in HCC cells through a similar or distinct mechanism(s).The conjugated bile salt, glycochenodeoxyocholic acid (GCDA), has been reported to activate PI3K/AKT and MAPK ERK1/2 signaling pathways in association with increased survival and proliferation of SEG-1 cells (a Barrett's adenoma cell line) [24,25]. However, the downstream survival substrate(s) is not yet identified. Here we demonstrate that GCDA-activated ERK1/2 induces Mcl-1 phosphorylation at T163, which stabilizes Mcl-1 protein to enhance its antiapoptotic function. Additionally, GCDA can also induce apurinic/apyrimidinic (AP) sites of DNA lesions. These properties of GCDA may contribute to the development of HCC and/or chemoresistance.

## Results

### GCDA induces activation of ERK1/2 and Mcl-1 phosphorylation leading to increased association with Bim in HepG2 cells

**Figure 1:**
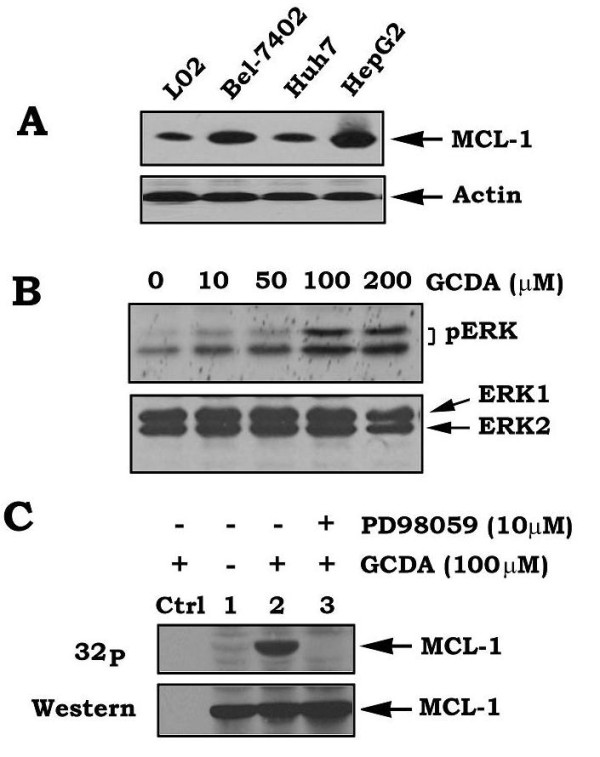
**GCDA activates ERK1/2 and induces Mcl-1 phosphorylation in HepG2 cells**. (**A) **Expression of Mcl-1 in various HCC cell lines and normal liver cell line L02 was analyzed by Western blot. **(B) **HepG2 cells were treated with increasing concentrations of GCDA for 30 min. Phosphorylation of ERK1/2 or total ERK1/2 was analyzed by Western blot using phospho-specific ERK or ERK1/2 antibodies, respectively. **(C) **HepG2 cells were metabolically labeled with ^32^P-orthophosphoric acid for 60 min and treated with GCDA (100 μM) in the absence or presence of PD98059 (10 μM) for 30 min. Mcl-1 was immunoprecipitated by using Mcl-1 antibody. Phosphorylation of Mcl-1 was determined by autoradiography. Western blot analysis was performed to confirm and quantify Mcl-1 protein. *Ctrl*, immunoprecipitation with normal rabbit IgG as a negative control.

Mcl-1 is a major antiapoptotic molecule of the Bcl2 family that is expressed in about 50% of HCC [1]. High levels of endogenous Mcl-1 were also observed in various HCC cell lines (Figure 1A). Thus, Mcl-1 may play a critical role in regulating survival and/or chemoresistance of HCC. The bile salt GCDA has been reported to activate ERK1/2 in SEG-1 cells [24]. To test whether GCDA can also activate ERK1/2 in HCC cells, HepG2 cells were treated with GCDA (100 μM) for 30 min. Phosphorylation of ERK1/2 was analyzed by Western blot using a phospho-specific ERK antibody. Results indicate that GCDA induces phosphorylation and activation of ERK1/2 in HepG2 cells in a dose-dependent manner (Figure 1B). Since ERK1 and ERK2 are physiologic Mcl-1 kinases [18], GCDA-activated ERK1/2 may phosphorylate Mcl-1 in HepG2 cells. To test this possibility, HepG2 cells were metabolically labeled with ^32^P-orthophosphoric acid and treated with GCDA in the absence or presence of the MEK inhibitor PD98059 for 30 min. Results indicate that GCDA potently stimulates phosphorylation of endogenous Mcl-1 in HepG2 cells (Figure 1C). Importantly, inhibition of MEK/ERK by PD98059 blocks GCDA-induced Mcl-1 phosphorylation (Figure 1C). These findings suggest that GCDA-stimulated Mcl-1 phosphorylation may occur through activation of ERK1/2, which may contribute to enhanced chemoresistance. To test the effects of GCDA on ERK1/2 and Mcl-1 in normal liver and other HCC cell lines, similar experiments were carried out in L02, Bel-7402, Huh7 and HepG2 cells. Results reveal that GCDA can also activate ERK/2 and induce Mcl-1 phosphorylation in normal liver L02 cells and various types of HCC cells (Additional file 1, Figure S1).

**Figure 2:**
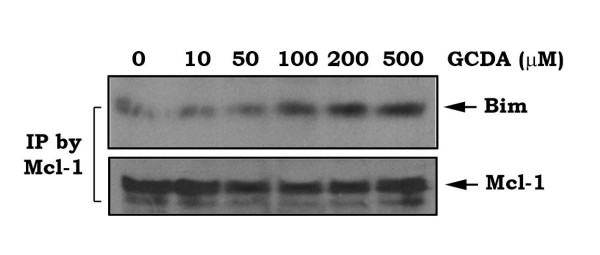
**GCDA promotes Mcl-1/Bim interaction**. HepG2 cells were treated with increasing concentrations of GCDA for 24h. A co-immunoprecipitation was performed using a Mcl-1 antibody. The Mcl-1-associated Bim and Mcl-1 were analyzed by Western blot.

Mcl-1 is thought to inhibit apoptosis by sequestering the proapoptotic BH3-only proteins (*i.e*. Bim) and/or binding the proapoptotic, multidomain Bak [10]. To test whether GCDA-induced Mcl-1 phosphorylation affects its ability to associate with Bim, HepG2 cells were treated with increasing concentrations of GCDA. A co-immunoprecipitation experiment was performed to measure the Mcl-1/Bim interaction. Results reveal that GCDA promotes Mcl-1 to associate with Bim in a dose-dependent manner (Figure 2), which may suppress the proapoptotic function of Bim to enhance survival and chemoresistance of HCC cells.

### GCDA stimulates Mcl-1 phosphorylation at T163 site in association with increased chemoresistance

**Figure 3:**
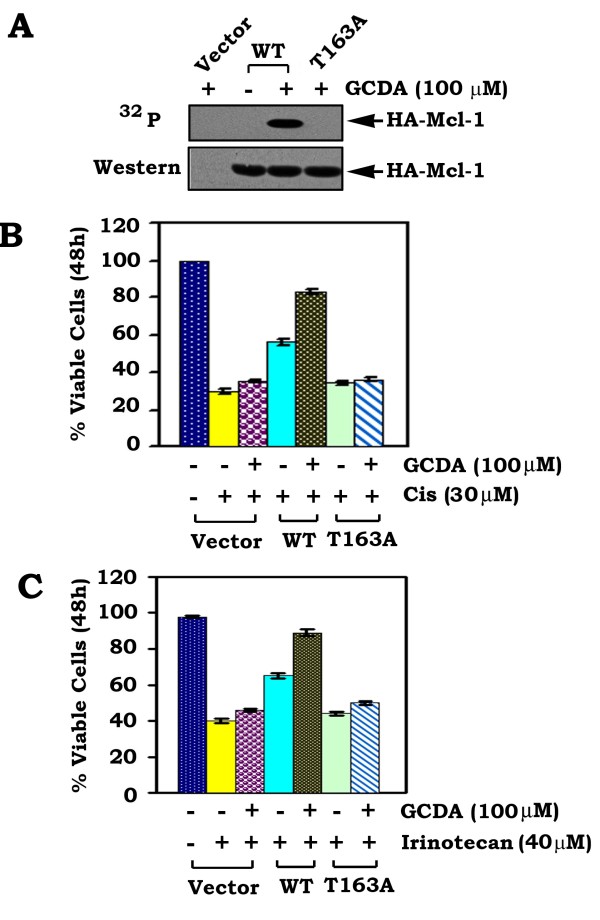
**GCDA induces Mcl-1 phosphorylation at T163 site, which is essential for GCDA-stimulated survival**. **(A) **Huh7 cells overexpressing exogenous HA-tagged WT, T163A Mcl-1 mutant or vector-only control cells were metabolically labeled with ^32^P-orthophosphoric acid and treated with GCDA for 30 min. Mcl-1 was immunoprecipitated using an HA antibody. Phosphorylation of Mcl-1 was determined by autoradiography. **(B) and (C) **Huh7 cells expressing WT or T163A Mcl-1 mutant were treated with cisplatin (30 μM) or irinotecan (40 μM) in the absence or presence of GCDA (100 μM) for 48 h. Cell viability was determined by analyzing annexin-V binding by FACS. Data represent the mean ± S.D. of three separate determinations.

ERK1 and ERK2 have been reported to phosphorylate Mcl-1 at T163 and enhance the antiapoptotic activity of Mcl-1 [18]. Because GCDA can activate ERK1/2, it is possible that GCDA may stimulate Mcl-1 phosphorylation at the T163 site. To test this, we have constructed HA-tagged WT and the non-phosphorylatable T163A Mcl-1 mutant. WT and the T163A Mcl-1 mutant were overexpressed in Huh7 cells that express relatively low levels of endogenous Mcl-1. Cells overexpressing HA-tagged WT, T163A or vector-only control were metabolically labeled with ^32^P-orthophosphoric acid and treated with GCDA (100 μM) for 30 min. HA-tagged Mcl-1 was immunoprecipitated using an HA antibody. Phosphorylation of exogenous HA-tagged Mcl-1 was analyzed by autoradiography. The same filter was then probed by Western blot using an HA antibody. Since GCDA induces phosphorylation of WT but not T163A Mcl-1 mutant (Figure 3A), GCDA-stimulated Mcl-1phosphorylation may occur exclusively at the T163 site. Importantly, GCDA prolongs survival of cells expressing WT but not the T163A Mcl-1 mutant following treatment with cisplatin or irinotecan (Figure 3BC). These findings provide genetic evidence that phosphorylation of Mcl-1 at T163 is essential for GCDA-induced survival of HCC cells.

### GCDA increases the half-life of Mcl-1 and disrupts its interaction with E3 ligase Mule in HepG2 cells

**Figure 4:**
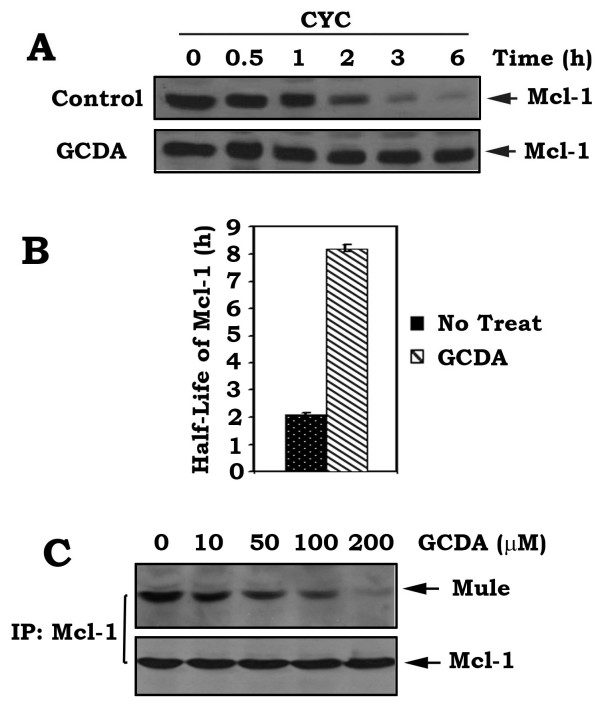
**Treatment of cells with GCDA disrupts Mule/Mcl-1 interaction and prolongs the half-life of Mcl-1**. **(A) **HepG2 cells expressing high levels of endogenous Mcl-1 were treated with 100 μg/ml cycloheximide 5 min prior to starting the indicated time course (*i.e*. 0 h, 0.5 h, 1 h, 2 h, 3 h, 6 h) in the absence or presence of GCDA (100 μM). Mcl-1 was analyzed by Western blot. **(B) **HepG2 cells were metabolically labeled with ^35^S-methionine. A classic pulse-chase experiment with the same time course as above (*i.e*. 0 h, 0.5 h, 1 h, 2 h, 3 h, 6 h) was carried out. The half-life of Mcl-1 was determined by electronic autoradiography. **(C) **HepG2 cells were treated with increasing concentrations of GCDA. A co-immunoprecipitation was carried out using an Mcl-1 antibody. The Mcl-1-associated Mule and Mcl-1 were analyzed by Western blot.

Mcl-1 has a short-term pro-survival function due to its short half-life. A previous report indicates that bile acids can increase total cellular Mcl-1 protein levels in KMBC cells, which is dependent upon activation of an EGFR/Raf-1 cascade [26]. To test whether GCDA-stimulated T163 phosphorylation of Mcl-1 affects its stability in HCC cells, the half-life of Mcl-1 was measured using cycloheximide blocking methods as described [27]. HepG2 cells were treated with 100 μg/ml cycloheximide in the absence or presence of GCDA (100 μM) for various times as indicated. Results reveal that GCDA significantly prolongs the half-life of Mcl-1 (Figure 4A). To more accurately evaluate the turnover rate of Mcl-1, we also used the classical ^35^S-methionine pulse-chase method as we previously described [28]. The ^35^S-methionine-labeled cells were washed and incubated in fresh methionine-replete RPMI medium 1640 in the absence or presence of GCDA (100 μM) for various times (*i.e*. 0 h, 0.5 h, 1 h, 2 h, 3 h and 6 h). The half-life (t_1/2_) of Mcl-1 was determined by electronic autoradiography. Consistently, exposure of cells to GCDA increases the half-life of Mcl-1 from 2.1 h to 8.2 h (Figure 4B). These data suggest that GCDA-prolonged half-life of Mcl-1 may lead to a long-term survival activity. Mule is a known Mcl-1 ubiquitin E3 ligase that can directly interact with Mcl-1 to facilitate Mcl-1 ubiquitination and degradation [29]. To test whether GCDA-induced Mcl-1 phosphorylation affects Mule/Mcl-1 association, HepG2 cells were treated with increasing concentrations of GCDA for 30 min. A co-immunoprecipitation was performed using an Mcl-1 antibody. Intriguingly, treatment of cells with GCDA results in dissociation of the Mule/Mcl-1 complex in a dose-dependent manner (Figure 4C), which may prevent Mcl-1 from Mule-mediated ubiquitination and degradation. This helps explain why GCDA significantly prolongs the half-life of Mcl-1 (Figure 4AB).

### GCDA reverses irinotecan-induced downregulation of pERK and Mcl-1 in association with prolonged survival of HepG2 cells

**Figure 5:**
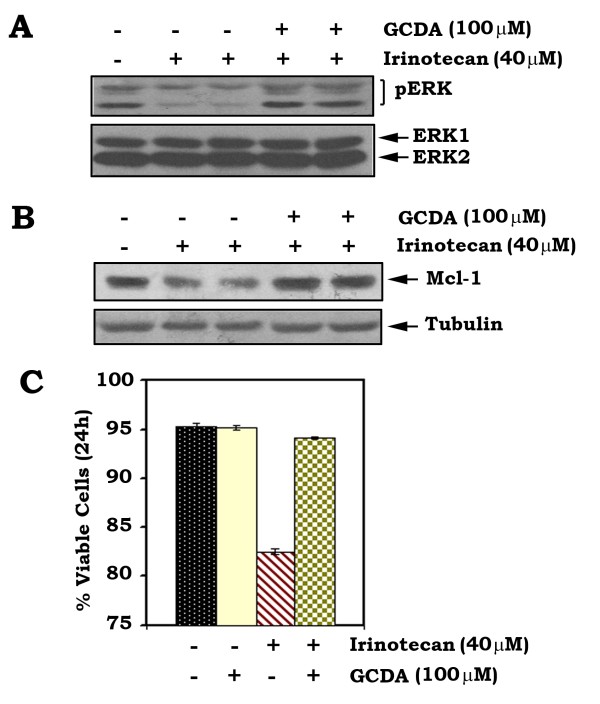
**GCDA reverses irinotecan-induced downregulation of pERK and Mcl-1 and enhances survival**. **(A) **and **(B) **HepG2 cells were treated with irinotecan in the absence or presence of GCDA for 24 h. The pERK, total ERK1/2 and Mcl-1 were analyzed by Western blot. **(C) **HepG2 cells were treated with irinotecan in the absence or presence of GCDA for 24 h. Cell viability was determined by analyzing annexin-V binding by FACS. Data represent the mean ± S.D. of three separate determinations.

Irinotecan is a topoisomerase-I inhibitor (*i.e*. camptothecin derivative, CPT11) which has been used as one of the first-line chemotherapeutic drugs for treatment of patients with advanced HCC [30,31]. Our findings indicate that treatment of HepG2 cells with irinotecan downregulates ERK phosphorylation in association with decreased Mcl-1 expression (Figure 5AB). Intriguingly, GCDA can reverse the negative effects of irinotecan on pERK and Mcl-1 expression (Figure 5AB). To further test whether GCDA affects the sensitivity of HepG2 cells to irinotecan, HepG2 cells were treated with irinotecan (*i.e*. 40 μM) in the absence or presence of GCDA (*i.e*. 100 μM) for 24 h. Results reveal that GCDA prolongs survival of HepG2 cells following irinotecan treatment, suggesting that GCDA may contribute to chemoresistance during HCC treatment (Figure 5C).

### GCDA induces AP sites of DNA lesions in HepG2 cells

Our findings show that GCDA can stimulate the survival signaling pathway (*i.e*. GCDA/ERK/Mcl-1). In addition to the ERK/Mcl-1 survival pathway, we also tested the effect of GCDA on DNA damage. HepG2 cells were treated with increasing concentrations of GCDA for 60 min. DNA damage was analyzed by Comet assay as described under "Experimental Procedures." Results reveal that GCDA induces DNA damage in a dose-dependent manner (Additional file 2, Figure S2A). Cells that have accumulated DNA damage appear as fluorescent "comets" with tails of DNA fragmentation or unwinding, whereas normal undamaged DNA does not migrate far from the origin (Additional file 2, Figure S2A). The Comet assay is able to detect SSBs, DSBs, and apurinic/apyrimidinic (AP) sites. Because GCDA can induce oxidative DNA damage [22,23], this may lead to the release of free bases from DNA, generating strand breaks with various sugar modifications and AP sites. To test whether exposure of cells to GCDA increases AP sites of DNA lesions, AP sites in genomic DNA were assessed using a DNA damage quantification (AP Site Counting) kit and analyzed using a microplate reader with a 650-nm filter. Results indicate that treatment of HepG2 cells with GCDA significantly enhances the AP sites in genomic DNA (Additional file 2, Figure S2B), which may partially neutralize its cell survival activity resulting from activation of ERK/Mcl-1. Based on the effects of GCDA on survival and AP sites of DNA lesions, a long-term exposure of cells to GCDA may result in an accumulation of DNA damage in living cells leading to genetic instability and/or tumorigenesis.

### Specific depletion of Mcl-1 blocks GCDA-stimulated survival of HepG2 cells following treatment with the chemotherapeutic drug cisplatin

**Figure 6:**
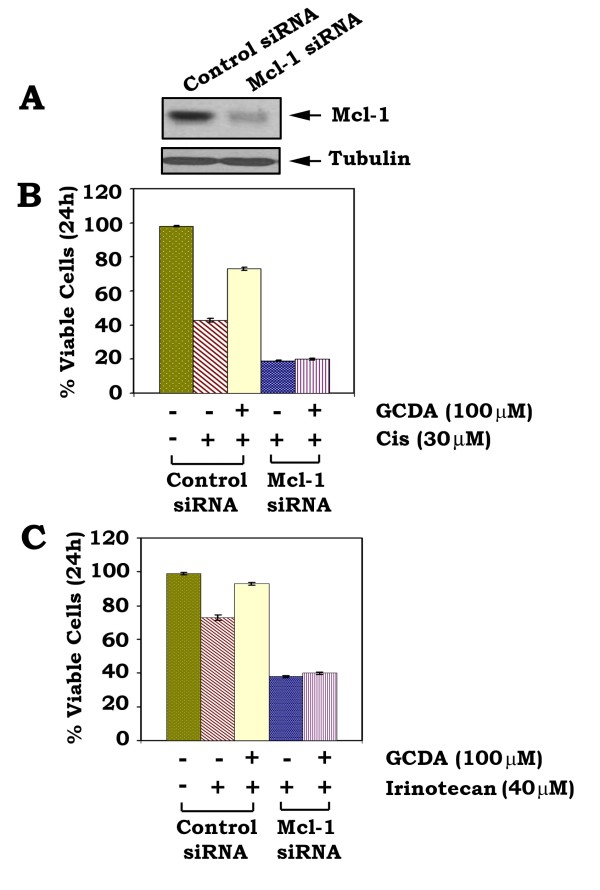
**Mcl-1 is essential for GCDA-induced chemoresistance in HepG2 cells**. **(A) **Mcl-1 siRNA (15 nM) or control siRNA (15 nM) was transfected into HepG2 cells using Lipofectamine 2000. After 24 h, the levels of Mcl-1 expression were analyzed by Western blot. **(B) **and **(C) **HepG2 cells expressing Mcl-1 siRNA or control siRNA were treated with cisplatin (30 μM) or irinotecan (40 μM) in the absence or presence of GCDA (100 μM) for 24 h. Cell viability was determined by analyzing annexin-V binding by FACS. Data represent the mean ± S.D. of three separate determinations.

To test whether Mcl-1 is a required target for GCDA-induced survival of human HCC cells, Mcl-1 siRNA or control siRNA was transfected into HepG2 cells. Results indicate that cells expressing Mcl-1 siRNA displayed a > 90% reduction of Mcl-1 protein expression (Figure 6A). Exposure of HepG2 cells without silencing of Mcl-1 prolongs survival of HepG2 cells following treatment with cisplatin or irinotecan (Figure 6BC; column 2 vs. column 3). By contrast, specific knockdown of Mcl-1 expression from HepG2 cells by RNAi blocks GCDA-prolonged survival of HepG2 cells following treatment with the chemotherapeutic drug cisplatin or irinotecan (Figure 6BC). GCDA had no additional survival effect on cells expressing Mcl-1 siRNA, suggesting that Mcl-1 may be a required target in GCDA-induced survival and/or chemoresistance of HepG2 cells.

## Discussion

Secretion of bile salts from the hepatocyte into bile is essential for the excretory function of the liver. Bile salts enter the duodenum to assist in the absorption of lipids and fat-soluble vitamins. Bile salts are passively and/or actively absorbed in the intestine and are transported back to the liver via the portal circulation. Bile salts are then taken up from the sinusoidal blood plasma by hepatocytes and their journey starts again [21]. Extrusion of bile salts from the hepatocyte is critical for liver cell survival and prevention of hepatocyte carcinogenesis because intracellular accumulation of bile salts not only causes DNA damage in hepatocytes ([22,23]; Figure S2) but also stimulates multiple cell survival and proliferation signaling pathways that may lead to tumorigenesis [24-26]. For example, patients with severe BSEP (*i.e*. the bile salt export pump) deficiency are at risk of developing HCC [21].

GCDA is one of the major bile salts in humans [32]. Bile salts and bile acids have been reported to be potential carcinogens [22,33] but the mechanism(s) involved remains elusive. On one hand, bile salts (*i.e*. GCDA) can stimulate cell survival and proliferation through activation of PI3K/AKT and MAPK pathways [24,25]. On the other hand, bile salts can also cause oxidative DNA damage, including accumulation of 8-hydroxydeoxyguanosine (8-OHdG) and DNA strand breaks [22,23]. Our findings suggest that GCDA-induced Mcl-1 phosphorylation at T163 may occur through activation of ERK1/2, which contributes to survival and chemoresistance of HCC cells (Figures. 1 and 3). Moreover, we also discovered that exposure of cells to GCDA significantly increases AP sites of DNA lesions (Figure S2). Based on these findings, we propose that, in addition to DNA damage, the mechanisms that promote cellular survival and proliferation may also be the critical steps in bile salt-initiated carcinogenesis.

Mcl-1 is a major antiapoptotic member of the Bcl2 family which has survival and oncogenic properties [34]. Intriguingly, Mcl-1 has been demonstrated to be a physiological downstream survival substrate of MAKP/ERK1/2 [12,18]. It is possible that the accumulated bile salts not only induce DNA damage in hepatocytes but also stimulate the ERK/Mcl-1 signaling pathway leading to hepatocyte survival and/or carcinogenesis. A recent report reveals that Mcl-1 is overexpressed in about 50% of HCC tissues [1], suggesting that Mcl-1 may be a valuable oncogenic and/or therapeutic target of HCC. Our findings reveal that GCDA potently stimulates Mcl-1 phosphorylation at the T163 site through activation of ERK1/2 (Figures. 1, 3 and S1). It is known that constitutive K-ras mutations lead to activation of a downstream signal cascade involving Ras/Raf/MEK/ERK1/2 [35], which may potentially enhance Mcl-1 phosphorylation. However, only a low incidence of K-ras mutations (~5%) could be observed in HCC patients [36,37]. By contrast, a higher incidence of K-ras mutations in patients with cholangiocarcinoma (*i.e*. 62~66%) has been observed [38,39].

Mcl-1 is a protein with a short half-life of about 30 min~3 h [20,40]. Thus, mechanisms that enhance the half-life of Mcl-1 are critical for its long-term survival activity. Our findings reveal that GCDA significantly prolongs the half-life (*i.e*. from 2.1 h to 8.2 h) in HepG2 cells (Figure 4). This increased Mcl-1 protein stability may result from GCDA-induced T163 site Mcl-1 phosphorylation because T163 site phosphorylation has been demonstrated to stabilize Mcl-1 in cells [18,20]. Mule has been identified as Mcl-1 ubiquitin E3 ligase that can induce polyubiquitination and degradation of Mcl-1 [29]. Our data indicate that GCDA can disrupt the Mcl-1/Mule interaction in cells (Figure 4C), suggesting that GCDA-induced T163 site phosphorylation may prevent Mcl-1 from Mule binding and Mule-mediated polyubiquitination and degradation. Furthermore, GCDA-induced Mcl-1 phosphorylation promotes Mcl-1 to interact with the BH3-only protein Bim (Figure 2), which should render Mcl-1 able to more efficiently suppress the proapoptotic function of Bim. These findings uncover the molecular mechanisms by which bile salts positively regulate survival and/or enhance chemoresistance of HCC cells. Since specific knockdown of Mcl-1 by RNAi blocks GCDA-induced survival of HepG2 cells following treatment with cisplatin or irinotecan (Figure 6), our findings indicate that Mcl-1 may be a required target for GCDA-induced survival of HCC cells.

In summary, our findings have provided strong evidence that GCDA prolongs survival of HCC cells in a mechanism that enhances the antiapoptotic function of Mcl-1 through T163 site phosphorylation. GCDA-induced Mcl-1 phosphorylation dissociates Mcl-1 from its E3 ligase binding, which enhances the half-life of Mcl-1 leading to its long-term survival function and/or chemoresistance of HCC cells. Therefore, manipulation of the antiapoptotic function of Mcl-1 by disrupting T163 site phosphorylation may represent a new strategy for treatment of hepatocellular carcinoma and/or other malignancies overexpressing Mcl-1.

## Methods

### Materials

Mcl-1, pERK, ERK1/2, Bim and tubulin antibodies as well as Mcl1 siRNA were purchased from Santa Cruz Biotechnology (Santa Cruz, CA). Anti-Mule antibody was purchased from Bethyl Laboratories, INC. (Montgomery, TX). The conjugated bile salt, glycochenodeoxyocholic acid (GCDA) was obtained from Sigma (St. Louis, Mo). PD98059 was purchased from EMD Chemicals, INC. (Gibbstown, NJ). All other reagents used were obtained from commercial sources unless otherwise stated.

### Cell lines, metabolic labeling, immunoprecipitation and Western blot

Hepatocellular carcinoma cell lines (HepG2, BeL-7402 and Huh7) and normal liver cell line L02 were obtained from the Cell Bank of Type Culture Collection of Chinese Academy of Sciences (Shanghai, China) and were maintained in DMEM medium with 10% fetal bovine serum (FBS) in a humidified cell culture incubator with 5% CO_2 _at 37°C. For measurement of Mcl-1 phosphorylation, cells were washed with phosphate-free RPMI medium and metabolically labeled with ^32^P-orthophosphoric acid for 60 min. After treatment with GCDA, cells were washed with ice-cold PBS and lysed in detergent buffer. Mcl-1 was immunoprecipitated and subjected to 10% SDS/PAGE, transferred to a nitrocellulose membrane and exposed to Kodak X-omat film at -80°C. Mcl-1 phosphorylation was determined by autoradiography. The same filter was then probed by Western blot as described previously [20].

### RNA interference

Human Mcl-1 siRNA (GAAGACCAUAAACCAAGAAtt) was purchased from Santa Cruz Biotechnology. HepG2 cells were transfected with Mcl1 siRNA using Lipofectin 2000 (Invitrogen). A control siRNA (nonhomologous to any known gene sequence) was used as a negative control. The levels of Mcl1 expression were analyzed by Western blot. Specific silencing of the targeted Mcl-1 gene was confirmed by at least three independent experiments.

### Comet assay

A single-cell gel electrophoresis (Comet Assay) kit was employed for evaluating DNA damage following the manufacturer's instructions (Trevigen, Inc., Gaithersburg, MD). Comet assay is an effective method for detection of DNA damage in cells. The principle of the assay is based upon the ability of denatured, cleaved DNA fragments to migrate out of the cell under the influence of an electric field, whereas undamaged DNA migrates slower and remains within the confines of the nucleoid when a current is applied. After treatment of cells with GCDA, cells in 1× PBS (Ca^2+^- and Mg^2+^-free) at 1 × 10^5^/ml were combined with molten LMAgarose (at 37 °C) at a ratio of 1:10 (v/v) and 75 μl immediately pipetted onto a CometSlide™. After a gentle cell lysis, samples were treated with alkali to unwind and denature the DNA and hydrolyze sites of damage. The samples were submitted to electrophoresis, stained with a fluorescent DNA intercalating dye (*i.e*. SYBR Green I nucleic acid), and visualized by a fluorescence microscope (Zeiss).

### AP Site Counting in Genomic DNA

AP sites of DNA lesions were analyzed as we described previously [41,42]. Briefly, genomic DNA was purified using a DNA isolation kit (Dojindo Molecular Technologies, Inc., Gaithersburg, MD). The number of AP sites was assessed using a DNA damage quantification (AP site counting) kit according to the manufacturer's instructions. Aldehyde reactive probe (ARP) reagent (*N*'-aminooxymethylcarbonylhydrazino-D-biotin) can react specifically with an aldehyde group that is the open ring form of the AP sites. After treating DNA containing AP sites with ARP reagent, AP sites are tagged with biotin residues. By using an excess amount of ARP, all AP sites can be converted to biotin-tagged AP sites. Standard ARP DNA and purified ARP-labeled sample genomic DNA was fixed on a 96-well plate with DNA binding solution. The number of AP sites in the sample DNA was determined by the biotin-avidin-peroxidase assay. The absorbance of the samples was analyzed using a microplate reader with a 650-nm filter. Each experiment was repeated three times, and the data represent the means ± S.D. of three determinations.

### Measurement of the Mcl-1 half-life

Cycloheximide half-life assay was performed as described [27]. Briefly, HepG2 cells were treated with 100 μg/ml cycloheximide 5 min prior to starting the indicated time course in the absence or presence of GCDA (100 μM). Cells were collected and lysed at the indicated times. Mcl-1 in total cell lysate was analyzed by Western blot. To more accurately quantify the turnover rate of Mcl-1, a classical ^35^S-methionine pulse-chase method was also employed. Cells were metabolically labeled with ^35^S-methionine for 60 min. The ^35^S-methionine-labeled cells were washed and incubated in fresh methionine-replete RPMI medium 1640 in the absence or presence of GCDA (100 μM) for various time points up to 12 h. ^35^S-labeled Mcl-1 was immunoprecipitated by using Mcl-1 antibody. The samples were subjected to SDS/10-20% PAGE. The half-life (*t*_1/2_) of Mcl-1 was determined by electronic autoradiography as described [28].

### Cell Viability Assay

Apoptotic and viable cells were detected using an ApoAlert Annexin-V kit (Clontech) according to the manufacturer's instructions. The percentage of annexin-V^low ^(*i.e*. viable) or annexin-V^high ^(*i.e*. apoptotic) cells was determined using the data obtained by fluorescence-activated cell sorter analysis as described [28]. Cell viability was also confirmed using the trypan blue dye exclusion.

## List of abbreviations

GCDA: glycochenodeoxycholate; HCC: hepatocellular carcinoma; ERK: extracellular signal-regulated kinase; BH: Bcl2 homology domain; S: serine, threonine, T; A: alanine; siRNA: small interfering RNA; RNAi: RNA interference; AP site: apurinic/apyrimidinic site; BSEP: bile salt export pump.

## Competing interests

The authors declare that they have no competing interests.

## Authors' contributions

Conceived and designed the experiments: XD, JZ and YZ; Performed experiments: ML, TW, JZ, JD and XD; Analyzed data: XD, JZ and ML; Wrote paper: XD, ML and JZ. All authors read and approved the final manuscript.

## Supplementary Material

Additional file 1 **Figure S1**. Effects of GCDA on phosphorylation of ERK1/2 and Mcl-1 in normal liver and various hepatocellular carcinoma cell lines. Click here for file

Additional file 2 **Figure S2**. GCDA induces DNA damage in HepG2 cells. Click here for file
