# Supplementary material for: Role of bile salt in regulating Mcl-1 phosphorylation and chemoresistance in hepatocellular carcinoma cells
Source: Mol Cancer. 2011 Apr 20;10:44. doi: 10.1186/1476-4598-10-44 (PMC3107804; doi:10.1186/1476-4598-10-44)
Supplement: Additional file 1 — Figure S1. Effects of GCDA on phosphorylation of ERK1/2 and Mcl-1 in normal liver and various hepatocellular carcinoma cell lines. [file 1476-4598-10-44-S1.PDF]

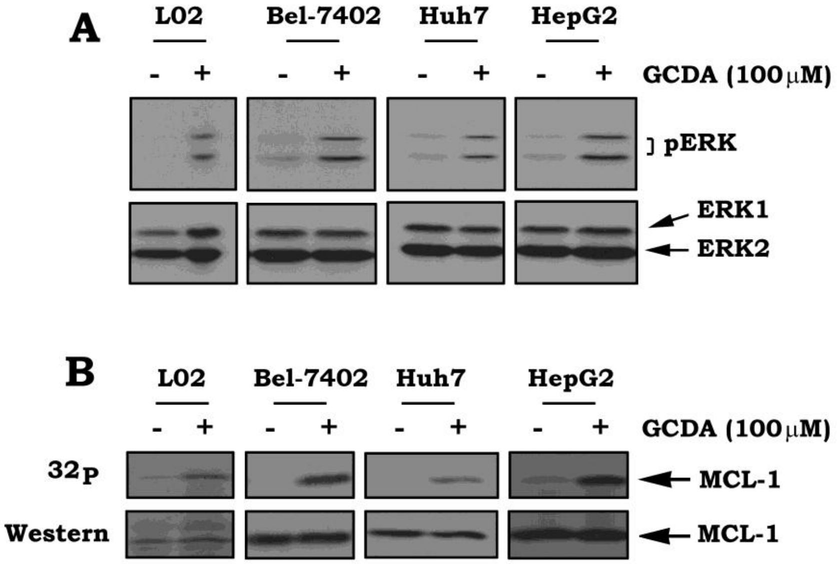

Figure S1. Effects of GCDA on phosphorylation of ERK1/2 and Mcl-1 in normal liver and various hepatocellular carcinoma cell lines. (A) L02, Bel-7402, Huh7 and HepG2 cells were treated with GCDA (100 μM) for 30 min. Phosphorylation of ERK1/2 or total ERK1/2 were analyzed by Western blot using phospho-specific ERK or ERK1/2 antibodies, respectively. **(B)** L02, Bel-7402, Huh7 and HepG2 cells were metabolically labeled with ^{32}P-orthophosphoric acid for 60 min and treated with GCDA (100 μM) in the absence or presence of PD98059 (10 μM) for 30 min. Mcl-1 was immunoprecipitated by using Mcl-1 antibody. Phosphorylation of Mcl-1 was determined by autoradiography. Western blot analysis was performed to confirm and quantify Mcl-1 protein.
