# Supplementary material for: Role of bile salt in regulating Mcl-1 phosphorylation and chemoresistance in hepatocellular carcinoma cells
Source: Mol Cancer. 2011 Apr 20;10:44. doi: 10.1186/1476-4598-10-44 (PMC3107804; doi:10.1186/1476-4598-10-44)
Supplement: Additional file 2 — Figure S2. GCDA induces DNA damage in HepG2 cells. [file 1476-4598-10-44-S2.PDF]

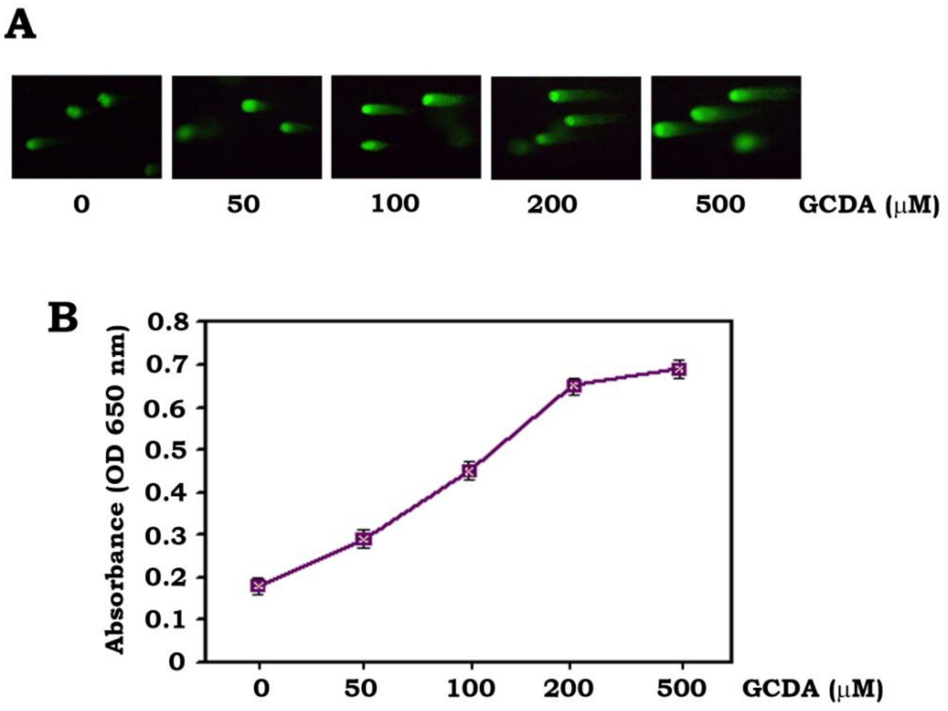

Figure S2. GCDA induces DNA damage in HepG2 cells. (A) HepG2 cells were treated with increasing concentrations of GCDA for 60 min. DNA damage was analyzed by Comet assay. **(B)** HepG2 cells were treated with increasing concentrations of GCDA for 60 min. AP sites of DNA lesions were analyzed using a DNA damage quantification (AP site counting) kit. Data represent the mean ± S.D. of three separate determinations.
